# Retraction Note: Activation of Wnt/β-catenin signalling promotes mesenchymal stem cells to repair injured alveolar epithelium induced by lipopolysaccharide in mice

**DOI:** 10.1186/s13287-022-02731-4

**Published:** 2022-02-03

**Authors:** Shi-xia Cai, Ai-ran Liu, Song Chen, Hong-li He, Qi-hong Chen, Jing-yuan Xu, Chun Pan, Yi Yang, Feng-mei Guo, Ying-zi Huang, Ling Liu, Hai-bo Qiu

**Affiliations:** 1 Department of Critical Care Medicine, Zhong-da Hospital, School of Medicine, Southeast University, 87 Dingjiaqiao Road, Nanjing, 210009 People’s Republic of China; 2 Department of Critical Care Medicine, The Affiliated Hospital of Qingdao University, 16 Jiangsu Road, Qingdao, 266003 People’s Republic of China; 3 State Key Laboratory of Natural Medicines, School of Life Science and Technology, China Pharmaceutical University, 24 Tongjiaxiang, Nanjing, 210009 People’s Republic of China

## Retraction Note: *Stem Cell Research & Therapy* (2015) 6:65 https://doi.org/10.1186/s13287-015-0060-y

The Editors-in-Chief have retracted this article. Concerns were raised about image overlap in Fig. 2A between NS + PBS/3 day and NS + PBS/14 day, and between LPS + mMSCs-Ctnnb1/3 day and LPS + mMSCs control/7 day. In response to these concerns, the authors provided original images, which contained further cases of overlap and duplication among images representing different samples. The Editors-in-Chief therefore no longer have confidence in the integrity of the data and results of this article.

All authors agree to this retraction.

